# The future of sequencing: convergence of intelligent design and market Darwinism

**DOI:** 10.1186/gb4168

**Published:** 2014-03-25

**Authors:** William J Greenleaf, Arend Sidow

**Affiliations:** 1 Department of Genetics, Stanford University School of Medicine, Stanford, CA 94305, USA; 2 Department of Pathology, Stanford University School of Medicine, Stanford, CA 94305, USA

## Abstract

A report on the Advances in Genome Biology and Technology meeting held in Marco Island, Florida, USA, on February 12–15, 2014.

## 

The annual Advances in Genome Biology and Technology (AGBT) meeting in Marco Island, Florida, is the pre-eminent venue for cutting edge, high-throughput sequencing technology and applications. As such, it is a hot ticket, and the meeting was oversubscribed with about 300 posters and talks, and more than 800 attendees, despite the awful travel conditions that ensnared many in the iced-in East Coast airports.

The incredible improvements in sequencing technology have been often noted, but the shocking pace of this progress bears yet another mention: simply put, the greater than six order-of-magnitude improvement in sequencing cost per base seen in the last 12 or so years is unprecedented in the history of the development of any technology. Much of this incredible progress was seeded by strategic investments by the National Institutes of Health’s (NIH) National Human Genome Research Institute (NHGRI). A stirring platform presentation from Jeff Schloss (NHGRI, NIH, USA) provided a historical backdrop for the current ‘perpetual revolution’ in sequencing by outlining the contributions of the NIH’s $100,000 and $1,000 genome initiatives. From 454 to the Polonator, from SOLiD to Helicos, from Ion Torrent to Advanced Liquid Logic, Jeff memorably recounted how the NHGRI-sponsored requests for application helped to drive the innovation that has powered the genomics revolution.

With Illumina’s sequencing technology now comfortably ensconced as a near monopoly, most of the talks focused on new frontiers in application of existing technologies. However, there were several notable presentations that focused on other sequencing technologies, underscoring that even Illumina’s powerful machines are not sufficient to cover all applications or use cases.

### Technologies: one of these things is not like the other?

Perhaps PacBio would have been well advised to have saved the fireworks it sent into the Florida skies in 2008 for this year. Several talks focused on how PacBio-based or PacBio-assisted methods result in higher quality assemblies or gene models than traditional Sanger or more recent Illumina-only approaches. For example, Dick McCombie (Cold Spring Harbor, USA) showed a new PacBio-only yeast genome assembly, David Wheeler (Baylor College of Medicine, USA) presented structural variant discovery in cancer, and Sean McGrath (Washington University School of Medicine, USA) discussed how cDNA-based PacBio reads greatly improve transcript annotation. If these applications are portents of the future, we may need to prepare for the coexistence of Illumina with a long-read technology for some years to come. But will this long-read technology come from PacBio or Oxford Nanopore?

Oxford Nanopore technology uses a protein nanopore and a synthetic membrane to measure conductance changes as single-stranded DNA is ratcheted through the pore. A base calling algorithm then interprets these changes in conductance to identify six-mer sequences present within the pore during the transit, thereby reconstructing the sequences of individual long molecules of DNA. Oxford Nanopore’s technology was presented by David Jaffe, leader of the genome computation group at the Broad Institute. As Jaffe repeatedly pointed out, the raw data on two bacterial genomes was generated by the Broad group and the base-calling was performed by Oxford Nanopore, who sent the reads back to Jaffe. Jaffe used the data to resolve ambiguities in Illumina-only assemblies. The reads provided by Oxford Nanopore reached an average of more than 5 kb, with the longest reads over 10 kb, and Jaffe showed a slide made by Oxford Nanopore wherein the read length distribution was precisely overlapping with the fragment length distribution of the library. (This is important because PacBio reads are on average much shorter than the fragments that are read.) The Oxford data had high error rates but more than 80% of the reads had perfect 50-mer sections. Thus, these relatively long, if error-prone, nanopore reads are a first glimpse of what may yet come as these methods and base-calling algorithms improve. Oxford Nanopore has clearly achieved a technical tour de force, with the potential to threaten PacBio’s dominance in the ultra-long-read field.

Is there room for more short-read technologies? Hesaam Esfandyarpour from Genapsys (USA) thinks so. He wants to put one or more of his lunchbox-sized devices into every single laboratory. The Genapsys system involves all-electronic detection of incorporation of nucleotides into DNA clonally amplified on magnetic beads. These beads are held above a regular array of electronic detectors. However, the presentation was nearly data-free, leaving the audience craving more specifics regarding the mechanism of signal generation, current throughput, cost, turn-around time, read length, and error rate for this concept.

### Sample preparation and analysis

Joshua Burton (University of Washington, USA) discussed analysis methods to take long-range sequence information encoded in Hi-C data to provide an ultra-long-range scaffold for the completion of *de novo* human genomes. The expected distribution of linked reads seen in Hi-C rapidly decays as a function of linear sequence distance in the genome. Using an algorithm named LACHESIS (after the Greek goddess who cut the threads of life), they use a combination of data, including Hi-C reads, to provide 99% accuracy in ordering and orienting fragments into chromosome groups for *de novo* human genome scaffolding.

Carlos Bustamante (Stanford University, USA) discussed a simple method for the enrichment of human DNA reads from ancient DNA samples that are often contaminated with environmental DNA. This method, whole genome in-solution capture (WISC), uses whole genome transcription with a fraction of biotynlated rNTPs to create baits for the entire genome. These baits can be used to enrich for human DNA, as well as to deplete human DNA in situations where bacterial or viral DNA is the target of interest.

### Applications: single cells, spatial sequencing and DNA modifications

The age of single cell sequencing has arrived, and a number of talks discussed methods and applications for DNA and RNA sequencing from individual cells, thereby lifting the veil on cell-to-cell variation hidden in the ensemble average. Aviv Regev (Broad Institute, USA) discussed recent work deciphering the gene expression variation in individual immune cells, and Steven Fodor (Cellular Research, USA) described methods for quantification of RNAs using molecular barcodes that allow absolute quantification of transcript levels and avoid distortions due to PCR bias.

Paul Blainey (Broad Institute, USA) presented microfluidic methods for generating libraries for whole genome sequences from hundreds of individual bacterial cells. These reusable devices include on-chip sieve valves and columns for the purification of samples. With sample preparation costs rapidly outstripping sequencing costs - especially for bacterial genomes - his goal is to generate an entire sequencing library from a bacterium for roughly $1 total sample preparation costs, providing a means for deep profiling the evolutionary landscape of related cells, or whole genome-level analysis of environmental organisms.

In a similar spirit to single cell sequencing, Patrik Ståhl (Karolinska Institute, Sweden) described a method to use sequencing to develop a rich spatial picture of gene expression, thereby harnessing the immense power of high-throughput sequencing to perform a kind of gene expression microscopy. His method uses a microarray of DNA to provide a spatial grid of DNA barcodes. These barcodes were used to capture a panel of RNAs, which were then sequenced using standard sequencing technology. After sequencing, the barcodes allowed confident assignment of sequenced transcripts to the location of origin within the tissue slice. These data thus provided a digital picture of the spatial organization of gene expression with near-cellular or subcellular resolution. However, it appeared from these data that gene expression levels were digital (for example, on or off) for each ‘pixel’ of the microarray matrix, opening an exciting future possibility of increased dynamic range.

Zak Wescoe from the Akeson laboratory at UCSC gave a fascinating talk about discriminating DNA modifications on the basis of conductance signals in a nanopore that is similar to Oxford Nanopore technology. The ‘adapter’ enzyme that threads strands into the hemolysin pore is phi29 DNA polymerase (as opposed to the helicase apparently used by Oxford Nanopore for sequencing). Wescoe tested oligonucleotide constructs with different cytosine modifications (methyl-, hydroxymethyl-, formyl-, carboxyl-) and was able to find signals that distinguished them from one another, adding to the potential application space of nanopore sensing.

### Conclusions

As the sun set on the Florida coast at the close of AGBT 2014, a few points appeared clear: first, a battle was raging, primarily between Oxford Nanopore and PacBio for the long-read sequencing market. Second, investigations of nucleic acids obtained from single cells have moved from proof-of-concept to becoming a true driver of biological discovery. Finally, as evidenced by the wealth of presentations dealing with impressive biological applications (which we do not have sufficient space to fully address here), sequencing has become a standard method for attacking an array of complex biological questions. In brief, by transforming biology into DNA space, then making billions of digital measurements in reasonable amounts of time for reasonable cost, high-throughput enables data sets that are well matched to the underlying biological complexity. Thus, sequencing technologies are not only rapidly bringing on the age of the ‘personalized genome’, but are also driving a revolution in biological sciences on the broadest scale.

### Abbreviations

kb: kilobase; NHGRI: National Human Genome Research Institute; NIH: National Institutes of Health.

### Competing interests

The authors declare that they have no competing interests.

